# Near-Complete Genome Sequence of a SARS-CoV-2 Variant B.1.1.7 Virus Strain Isolated in Kazakhstan

**DOI:** 10.1128/mra.00619-22

**Published:** 2022-08-23

**Authors:** Bekbolat Usserbayev, Kunsulu Zakarya, Lespek Kutumbetov, Mukhit Orynbaуev, Kulyaisan Sultankulova, Yergali Abduraimov, Balzhan Myrzakhmetova, Kuandyk Zhugunissov, Aslan Kerimbayev, Aibarys Melisbek, Meiіrzhan Shirinbekov, Saken Khaidarov, Asankadyr Zhunushov, Yerbol Burashev

**Affiliations:** a Research Institute for Biological Safety Problems (RIBSP), Gvardeyskiy, Kazakhstan; b Faculty of Biology and Biotechnology, Al-Farabi Kazakh National University, Almaty, Kazakhstan; c Institute of Biotechnology of the National Academy of Sciences of the Kyrgyz Republic, Bishkek, Kyrgyzstan; Queens College CUNY

## Abstract

This research describes the genome sequence of severe acute respiratory syndrome coronavirus 2 (SARS-CoV-2) obtained from a patient with symptoms of coronavirus disease 2019 (COVID-19) who was infected in the Republic of Kazakhstan. Strain SARS-CoV-2/human/KAZ/Britain/2021 consists of 29,815 nucleotides and belongs to lineage B.1.1.7, according to the Pangolin COVID-19 database.

## ANNOUNCEMENT

An outbreak of a new type of severe acute respiratory syndrome coronavirus (SARS-COV-2) emerged in December 2019 in Wuhan City (Hubei Province, People’s Republic of China) (1). According to the taxonomic classification, SARS-CoV-2 belongs to the subgenus *Sarbecovirus*, genus *Betacoronavirus*, and family *Coronaviridae* (2–5).

The first human cases of coronavirus infection COVID-19 (coronavirus disease 2019) were recorded in the Republic of Kazakhstan (RK) in mid-March 2020 (6). According to World Health Organization data, on 14 June 2022, a total of 1,395,068 confirmed cases were registered in the Republic of Kazakhstan, resulting in 19,017 deaths (7). According to the Global Initiative on Sharing Avian Influenza Data (www.gisaid.org), 12 different clades of the SARS-COV-2 virus are currently active worldwide. Due to the rapid spread of new coronavirus infections, it is necessary to study the genetic properties of the viruses in order to create diagnostic drugs and vaccines. In order to fully characterize the viral genome sequence, molecular genetic studies were carried out using Sanger sequencing technology.

Strain SARS-CoV-2/human/KAZ/Britain/2021 was obtained from the Scientific and Practical Center for Sanitary and Epidemiological Expertise and Monitoring branch of the National Center for Public Health, a republican state enterprise on the right of economic use of the Ministry of Health of the Republic of Kazakhstan. Nucleic acids were extracted from the test sample using a QIAamp viral RNA minikit (Qiagen, Germany) according to the manufacturer’s protocol. Reverse transcription was performed using the SuperScript VILO cDNA synthesis kit (Invitrogen, USA). For amplification to cover the entire genome of the virus, 65 primer pairs were designed using the online program Primer-BLAST (http://www.ncbi.nlm.nih.gov/tools/primer-blast) in order to generate amplicons ranging in size from 610 to 770 bp; each of the designed primers overlap each other by about 100 bp. These primers were subjected to PCR and visualized using 1.5% agarose gel electrophoresis (Sigma, USA). The resulting whole PCR products were purified using the PureLink PCR purification kit (Thermo Fisher Scientific, USA). These purified amplicons were sequenced using the Sanger dideoxy method using an AB3130xl (Hitachi Applied Biosystems) 16-capillary genetic analyzer autosequencer with the BigDye Terminator 3.1 cycle sequencing kit (ABI, Foster City, CA, USA). Raw chromatograms were collected using Sequencher version 5 (Gene Codes Corp.). To assemble the genome, the sequences were aligned using BioEdit version 7.2.5. Phylogenetic analysis was performed using MEGA X (8).

**FIG 1:**
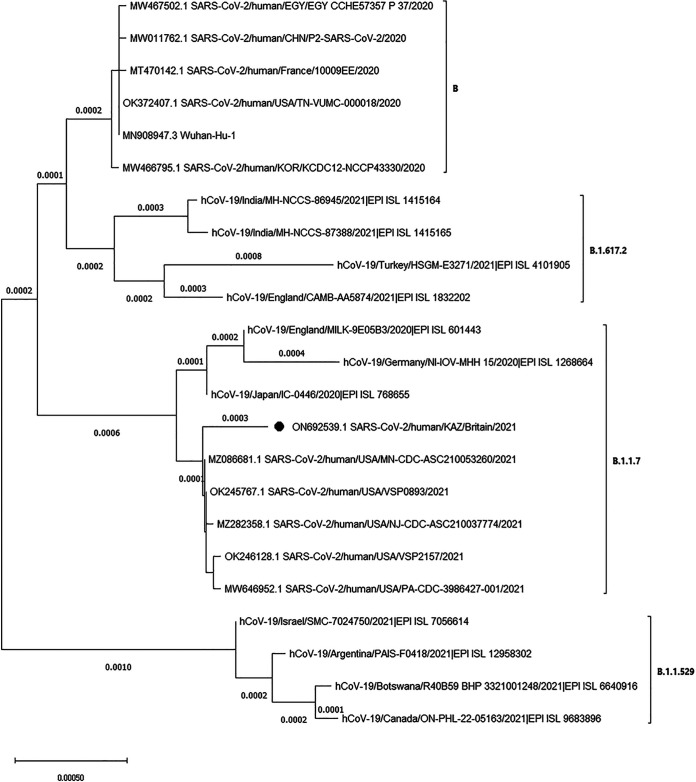
Phylogenetic analysis of isolate SARS-CoV-2/human/KAZ/Britain/2021, 11 global strains belonging to lineages B.1.617.2, B.1.17, and B.1.1.529 obtained from the GISAID database (https://www.gisaid.org), and 11 global strains belonging to lineages B and B.1.17 obtained from the NCBI database (https://www.ncbi.nlm.nih.gov/). Strain SARS-CoV-2/human/KAZ/Britain/2021 (black circle) shares a common ancestor with several U.S. isolates. Phylogenetic analysis was performed using MEGA X. Here, the *x* axis represents the scale of the tree.

The assembled complete genome sequence of strain SARS-CoV-2/human/KAZ/Britain/2021 is 29,815 nucleotides long, with a GC content of 38%. The resulting sequence was analyzed using the Pangolin COVID-19 database (https://pangolin.cog-uk.io) and found to belong to lineage B.1.1.7. The amino acid mutations were compared with a reference sequence, and the results are presented in Table 1. Phylogenetic analysis showed that the studied viral genome and several U.S. isolates have a common ancestor (Fig. 1).

**TABLE 1:** Mutations of isolate SARS-CoV-2/human/KAZ/Britain/2021 compared with the reference sequence Wuhan-Hu-1 SARS-CoV-2^*a*^

Protein	Amino acid location	Reference amino acid	Change in amino acids
ORF1ab	475	I	L
	1001	T	I
	1708	A	D
	2230	I	T
	2259	M	I
	3644	L	F
	3675	del S	
	3676	del G	
	3677	del F	
	4619	P	L
	4715	P	L
	6714	P	L
	6984	H	R
S	69	del H	
	70	del V	
	145	del Y	
	501	N	Y
	570	A	D
	614	D	G
	681	P	H
	716	T	I
	982	S	A
	1118	D	H
ORF 3a	149	W	L
N	3	D	L
	203	R	K
	204	G	R
	235	S	F

a GenBank accession number NC_045512.

### Data availability.

The complete nucleotide sequence of strain SARS-CoV-2/human/KAZ/Britain/2021 was deposited on 7 June 2022 at the National Center for Biotechnology Information under GenBank accession number ON692539.1.
